# Caprine Arthritis Encephalitis Virus Is Associated with Renal Lesions

**DOI:** 10.3390/v13061051

**Published:** 2021-06-01

**Authors:** Brian G. Murphy, Diego Castillo, Asli Mete, Helena Vogel, Dayna Goldsmith, Marietta Barro, Omar Gonzales-Viera

**Affiliations:** 1 Department of Pathology, Microbiology and Immunology, School of Veterinary Medicine, University of California, Davis, CA 95616, USA; castillodiego@yahoo.com (D.C.); hvogel@ucdavis.edu (H.V.); mvbarro@ucdavis.edu (M.B.); 2 California Animal Health and Food Safety Laboratory System, Davis Lab, 620 W Health Science Dr, Davis, CA 95616, USA; amete@ucdavis.edu (A.M.); gonzalesviera@ucdavis.edu (O.G.-V.); 3 Faculty of Veterinary Medicine, University of Calgary, Calgary, AB T2N 1N4, Canada; dayna.goldsmith@gmail.com

**Keywords:** SRLV, CAEV, TFBS, lentivirus, goat, promoter, kidney, interstitial nephritis

## Abstract

Caprine arthritis encephalitis virus (CAEV) is a monocyte/macrophage-tropic lentivirus that primarily infects goats resulting in a well-recognized set of chronic inflammatory syndromes focused on the joint synovium, tissues of the central nervous system, pulmonary interstitium and mammary gland. Clinically affected animals generally manifest with one or more of these classic CAEV-associated tissue lesions; however, CAEV-associated renal inflammation in goats has not been reported in the peer-reviewed literature. Here we describe six goats with chronic, multisystemic CAEV infections in conjunction with CAEV-associated renal lesions. One of the animals had CAEV antigen-associated thrombotic arteritis resulting in infarction of both the kidney and heart. These goats had microscopic evidence of inflammatory renal injury (interstitial nephritis) with detectable renal immunolabeling for CAEV antigen in three of six animals and amplifiable proviral sequences consistent with CAEV in all six animals. Cardiac lesions (vascular, myocardial or endocardial) were also identified in four of six animals. Within the viral promoter (U3) region, known transcription factor binding sites (TFBSs) were generally conserved, although one viral isolate had a duplication of the U3 A region encoding a second gamma-activated site (GAS). Despite the TFBS conservation, the isolates demonstrated a degree of phylogenetic diversity. At present, the clinical consequence of CAEV-associated renal injury is not clear.

## 1. Introduction

Caprine arthritis encephalitis virus (CAEV) is a lentivirus in the family Retroviridae that infects goats and is closely related to maedi-visna virus (MVV) of sheep [1,2]. As a result of their genetic similarities, these lentiviruses are collectively referred to as small ruminant lentiviruses or SRLVs. CAEV is a monocyte/macrophage-tropic virus that is not associated with immunodeficiency, generally infects dairy goats and was first reported as a retrovirus in 1980 [3,4]. Since this time, multiple studies have focused on defining the gross and microscopic lesions of naturally occurring and experimental SRLV infections in small ruminants [5,6,7]. CAEV is known to cause progressive inflammatory syndromes in a variety of tissues including the joint synovium in animals older than 12 months of age (caprine arthritis) or, less often, encephalomyelitis in 2–4-month-old goat kids. CAEV infection can also result in interstitial pneumonia or indurative mastitis (“hard udder”), lesions similar to those observed in sheep infected with MVV [8]. Histologically, these lesions are characterized by interstitial mononuclear infiltration often featuring lympho-follicular hyperplasia and fibrosis [9,10,11].

CAEV transcripts, in the absence of inflammatory lesions, have been reported in the renal tubular epithelium of goats naturally infected with CAEV (in situ hybridization) [12]. In these animals, CAE viral transcripts were also identified in a wide variety of tissues including the brain, spinal cord, lung, joint synovium, mammary gland, liver, spleen, lymph node, thyroid follicles and intestinal enterocytes. In sheep, inflammatory renal lesions associated with the deposition of MVV antigen and amplifiable proviral SRLV DNA have been reported in nine animals naturally infected with SRLVs [13]. These sheep had lesions consistent with interstitial nephritis characterized by infiltrates of lymphocytes, plasma cells and macrophages within the renal interstitium. Lymphoid follicle formation within the interstitium, a feature of SRLV pneumonia, was also reported in the renal tissues of these animals. The affected sheep in this study also had SRLV-associated pulmonary lesions (interstitial pneumonia).

Here we describe the lesional features and their association with CAEV proviral DNA and viral antigen deposition in six goats necropsied at the California Animal Health Food Safety Laboratory (CAHFS), Davis branch, and the University of California, Davis, Veterinary Medical Teaching Hospital (VMTH). In addition to renal lesions, these goats had multisystemic CAEV inflammatory syndromes affecting the joint synovium, lung, mammary gland and/or brain. Four of the six animals also had histological evidence of cardiac lesions. Despite the frequent occurrence of SRLVs and long-term investigation, CAEV-associated renal inflammation and SRLV-associated arterial lesions have not been previously reported in the peer-reviewed literature.

## 2. Materials and Methods

### 2.1. Animals

All of the goats included in this study were derived from farms/flocks in Central and Northern California, USA. A database search was conducted using the following keywords: goat, caprine arthritis encephalitis virus, CAE and small ruminant lentivirus. Goats A, E and F were identified prospectively as a CAHFS case in 2019 (A) or UC Davis VMTH cases in 2020 (E) or 2021 (F). Goats B and C were identified retrospectively from amongst all the caprine cases submitted to the CAHFS Laboratory System from 1990 to 2019 (801 animals total). Goat D was identified retrospectively from the UC Davis VMTH database using the same search terms defined above.

### 2.2. Pathology and Immunohistochemistry

Goats A, B and C were serologically tested for antibodies to SRLVs using the Small Ruminant Lentivirus Antibody Test Kit, cELISA (VMRD, Pullman, Washington) and were subsequently euthanized for quality of life issues. Goat D had a one-year history of progressive lameness affecting the left stifle and right carpal joints. Due to a deterioration in quality of life, euthanasia was elected. Goat E was treated for respiratory distress and pyrexia for 12 days using antibiotics and corticosteroids. As a result of progressive clinical deterioration in the face of therapy, euthanasia was recommended (goats E and F). Goats D and E were serologically tested with the VMRD cELISA postmortem using extracellular fluid obtained from frozen/thawed splenic tissue or serum, respectively. Goats A–E were adult does while goat F was a wether.

A complete necropsy was performed for each animal and all gross abnormalities were recorded. A complete set of tissues including lung, joint synovium, mammary gland, brain, heart and kidney were obtained for ancillary diagnostic studies. Tissues were immediately fixed in 10% buffered formalin for a minimum of 24 h, trimmed, embedded in paraffin and routinely processed for histological examination. Select tissues with lesions consistent with CAEV-associated inflammation were further evaluated by an immunohistochemistry (IHC) assay to detect CAEV antigen (MAb CAEV-63, CAEV-Co IgG1, CAEP10A1, VMRD, Pullman, WA). All of the immunohistochemical stains were performed at the CAHFS Davis laboratory. Each IHC stain was performed in parallel with known CAEV-positive caprine control tissue and an irrelevant isotype-control antibody as the negative control.

### 2.3. DNA Isolation, Amplification and Sequencing

Genomic DNA (proviral DNA) was isolated from either formalin-fixed paraffin-embedded (FFPE) scrolls of caprine tissue using QIAGEN’s QIAamp DNA FFPE Tissue Kit or from fresh-frozen tissue using QIAamp DNA Mini or Blood Mini Kit following the manufacturer’s protocol. The entire 3′ CAEV U3 region was amplified using a nested PCR strategy with recombinant Taq DNA Polymerase (Invitrogen, Waltham, MA, USA) following the manufacturer’s instructions for a 50 μL volume reaction. Primers that flank the 3′ CAEV U3 region, Rev_for_ (5′-CTG ACG ATG GGA ATC TGG ATA AAT GG), and R region of the long terminal repeat, R2_rev_ (5′-CTC GGT ACC TCC TCG GAG AGG AGA G), were used for a first round of amplification (456 nucleotide amplicon) under the following cycling conditions: 95 °C for 2 min; 25 cycles of 95 °C for 15 s, 56 °C for 30 s and 72 °C for 30 s; and a final extension step at 72 °C for 5 min. A second round of amplification was performed under the same cycling conditions with the substitution of 50 cycles instead of 25, using 5 μL of the first PCR reaction along with the primers CAEV_nested_ LTR_for_ (5′-TAA ATG GAM RGC KTG GAG AAC ACC WC) and CAEV_nested_ LTR_rev_ (5′-CTA GGA GSR MST CTC CYA GAA CTC) resulting in an amplicon of ~359 nucleotides that included the entire 3′ CAEV U3 region. All of the primer binding sites were located outside of the U3 region. Resulting amplicons from both the primary and nested PCR were screened on an agarose gel for size. A water template negative control was run in parallel with each PCR reaction set.

The nested PCR products of goats A, D and E were directly sequenced from both the 5′ and 3′ ends using the amplifying primers, CAEV_nested_ LTR_for_ and CAEV_nested_ LTR_rev_. Prior to direct sequencing, PCR amplicons were concentrated and purified using Zymo Research’s DNA Clean and Concentrator Kit (goat A) or with Amicon Ultra-0.5 Centrifugal Filter Unit (goats D and E). The consensus sequence was based upon both the forward and reverse sequences. The PCR amplicons of goats B, C and F were ligated into the pCR 2.1 plasmid using Invitrogen’s TA Cloning Kit and transformed into One Shot TOP10 Chemically Competent *E. coli* (ThermoFisher Scientific, Waltham, MA, USA). Three white bacterial colonies from each transformation reaction were amplified overnight in 5 mL of LB broth containing 100 μg/mL carbenicillin. Bacterial plasmids were purified using ZymoPURE Plasmid Miniprep Kit (Zymo Research, Irvine, CA) and were submitted for sequencing at the UC Davis College of Biological Sciences UCDNA Sequencing Facility. Amplicon sequences were digitally trimmed to include the entire U3 region and were aligned using MacVector software. A phylogenetic dendrogram of CAEV U3 sequences amplified from various tissues (mammary gland, lung, brain/spinal cord, joint synovium and kidney) of CAEV-infected goats in North America [14] was created using 48 sequences and MacVector software (nucleic acid alignment, MacVector 14.0, Apex, North Carolina) [14,15,16].

## 3. Results

### 3.1. Pathology

**Figure 1:**
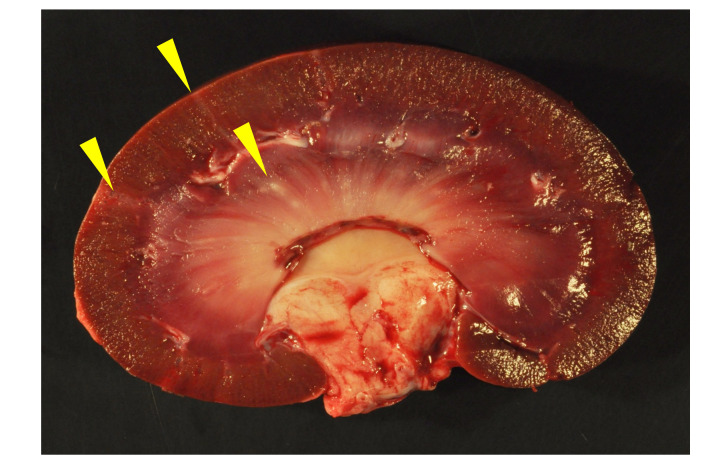
Gross image of kidney, goat D. Pale, linear radiating streaks are evident in the renal cortex and medulla (interstitial nephritis and fibrosis, yellow arrowheads).

All of the goats in this study were determined to be serologically positive for SRLV infection except for goat F (serum not available). Gross lesions were identified in the synovial joints (caprine arthritis), lungs (interstitial pneumonia), brain and/or mammary glands of multiple animals consistent with multisystemic CAEV inflammatory syndromes (Table 1). In addition to lesions typically attributable to the quartet of CAEV inflammatory syndromes, goat A had multiple pale wedge-shaped regions within the renal cortex and medulla of both kidneys which were interpreted as infarcts. The kidneys of goats B and D had multiple, approximately 1 mm diameter pale foci within the renal cortex and/or medulla (Figure 1). No gross changes were identified in the renal tissues of goats C, E and F. The only grossly evident cardiac lesions were aortic valvular endocarditis in goat C and pericardial effusion in goat D. Tissue preservation for goats B, C, D, E and F was considered to be excellent, while tissues from goat A were moderately autolyzed.

**Figure 2:**
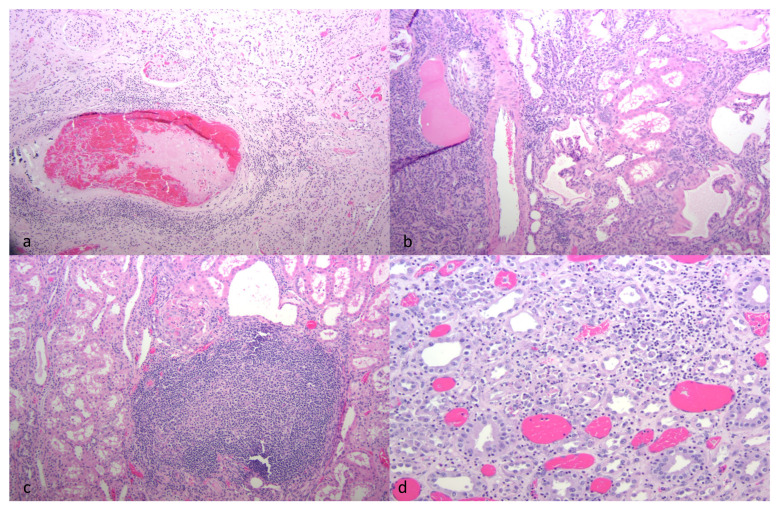
Histological features of CAEV-associated renal lesions (HE-stained sections). (**a**) A large artery at the corticomedullary junction has a luminal thrombus and is cuffed by moderate numbers of lymphocytes, (thrombotic arteritis, goat A). (**b**) The renal interstitium is expanded by large numbers of mononuclear lymphocytes and plasma cells (interstitial nephritis, goat B). (**c**) The renal cortex has an organized lymphoid follicle expanding the interstitium of goat B. (**d**) The medullary interstitium is infiltrated by moderate numbers of lymphocytes (interstitial nephritis, goat D).

**Figure 3:**
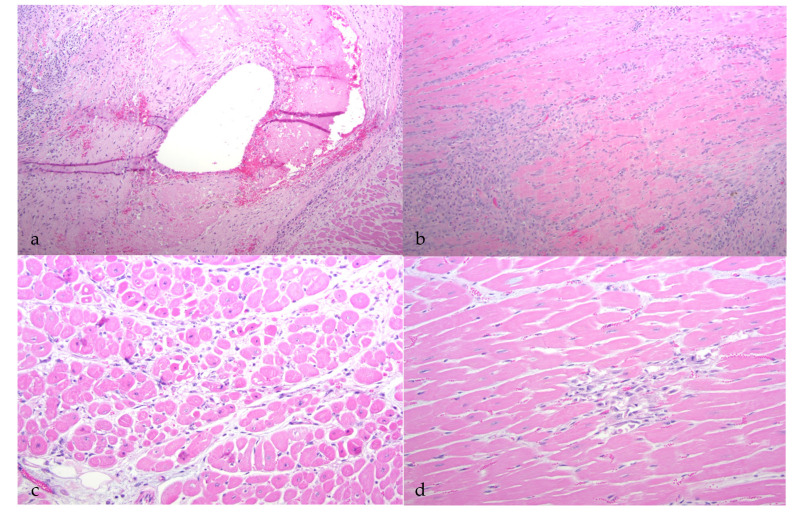
Histological features of CAEV-associated cardiac lesions (HE-stained sections). (**a**) A large artery within the myocardium has luminal and mural fibrin deposition and periarteriolar inflammation (thrombotic arteritis, goat A). (**b**) The myocardium has interstitial fibroplasia/fibrosis and lymphocytic inflammation (fibrosing myocarditis, goat C). (**c**) Cardiomyocytes are multifocally atrophied and have pyknotic nuclei (myocardial necrosis, goat D). (**d**) The myocardial interstitium has a focal loss of cardiomyocytes with interstitial fibroplasia (goat D).

**Figure 4:**
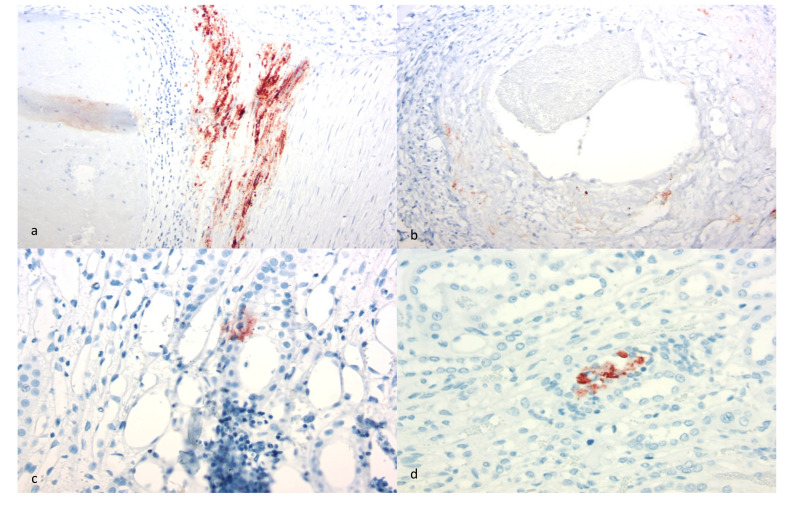
Immunohistological features of CAEV-associated cardiac and renal lesions (SRLV IHC). (**a**) Abundant CAEV antigen is present (brown staining granules) within the arterial tunica media (heart, goat A). (**b**) Moderate CAEV antigen is present within the arterial tunica media (kidney, goat A). (**c**) Focal CAEV antigen deposition is present within the tubular epithelium of the renal medulla (kidney, goat C). (**d**) Focal CAEV antigen is present within the tubular epithelium in the renal medulla (kidney, goat E).

Microscopically, the renal cortical interstitium of goat A was multifocally infiltrated by aggregates of lymphocytes and fibrous tissue consistent with a diagnosis of interstitial nephritis and fibrosis. In addition, the tunica media of multiple large muscular arteries within the renal cortex was expanded and disrupted by infiltrating lymphocytes and fibrin aggregate deposition consistent with lymphocytic fibrinonecrotizing arteritis. Several of the inflamed arteries had luminal fibrin thrombi and associated infarction (Figure 2a). The myocardium also had fibrinous arteritis lesions and luminal thromboses associated with myocardial infarcts (Figure 3a,b). Infarcted regions of the myocardium were replaced with fibrous tissue. Abundant CAEV antigen was detected in both the renal and myocardial arteritis lesions using IHC (Figure 4a,b).

In goat B, the renal cortical interstitium was multifocally infiltrated by large numbers of lymphocytes with fibrous tissue deposition consistent with a diagnosis of severe interstitial nephritis (Figure 2b). Interstitial inflammation also extended into the subjacent medulla. Affected cortical regions also demonstrated tubular atrophy, proteinuria, glomerular sclerosis and cystic glomeruli. In some regions, infiltrating lymphocytes were aggregated into lymphoid follicle-like structures (Figure 2c). CAEV antigen was not detected in the kidney using IHC. In the heart, minimal foci of lymphocytic myocarditis were identified.

In goat C, the renal medullary interstitium and vascular hilus were multifocally infiltrated by aggregates of lymphocytes (tubulointerstitial nephritis); areas of inflammation also demonstrated tubular necrosis. Scattered tubules had proteinuria and scattered glomeruli had small numbers of neutrophils within the glomerular capillaries. CAEV antigen was identified in a few endothelial cells and medullary tubules using IHC (Figure 4c). In the heart, the myocardial interstitium was multifocally altered by fibrous tissue and infiltrating lymphocytes and macrophages (myocarditis, Figure 3b). Atrioventricular valves were markedly disrupted by necrosis, fibrosis, aggregates of fibrin, hemorrhage and infiltrating lymphocytes and macrophages (valvular endocarditis). IHC performed on the myocardial tissue was negative.

Goat D had multifocal regions of interstitial inflammation and tubular necrosis within the renal medulla featuring lymphocytes and neutrophils (Figure 2d). CAEV antigen was not detected in the renal tissue by IHC. In the heart, multifocal regions of the myocardium were characterized by degeneration, necrosis and loss of cardiomyocytes. Necrotic myocardial foci were replaced with fibrous tissue and small numbers of infiltrating macrophages (Figure 3c,d). IHC was not performed on the heart.

Goat E had multifocal infiltration of the renal cortical and medullary interstitium with small numbers of lymphocytes and plasma cells. Inflammatory cells were often concentrated around veins. Multifocal glomerular capillaries also had small numbers of neutrophils. CAEV antigen was multifocally identified in the epithelial cells lining the medullary tubules and within luminal macrophages and necrotic debris using IHC (Figure 4d). The heart was not examined histologically (not collected in formalin).

Goat F had multifocal infiltration of the renal cortical interstitium with moderate numbers of lymphocytes and plasma cells. The interstitium was further expanded by a moderate amount of fibrous tissue. Multifocal glomerular capillaries also had small numbers of neutrophils (glomerulitis). CAEV antigen was not detected in the kidney with IHC. The heart was considered to be histologically normal.

### 3.2. PCR and Sequencing

**Figure 5:**
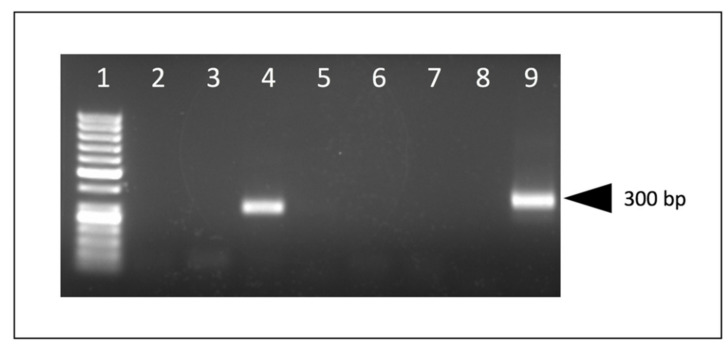
Nested PCR, agarose gel electrophoresis. An appropriate-sized PCR amplicon is present in samples derived from the kidney of goat A. Molecular weight markers (lane 1), kidney (positive, lane 4), other caprine tissue samples (negative, lanes 2, 3, 5, 7), water template (negative control, lanes 6 and 8), plasmid DNA (positive control, lane 9).

**Figure 6:**
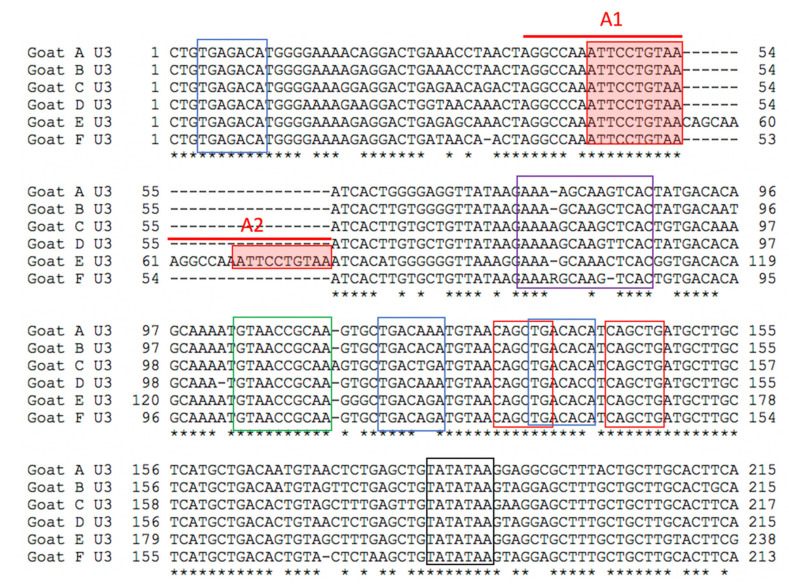
Alignment of the six CAEV U3 sequences derived from renal tissues of goats A, B, C, D, E and F. The two A blocks (A1 and A2, red bars) containing the GAS (filled red boxes) are indicated. Other transcriptional factor binding sites are also indicated—AP-1 (3 blue boxes), TAS (purple box), AML (green box), AP4 (2 red boxes) and TATA (black box). Consensus nucleotide positions are indicated with an asterisk.

**Figure 7:**
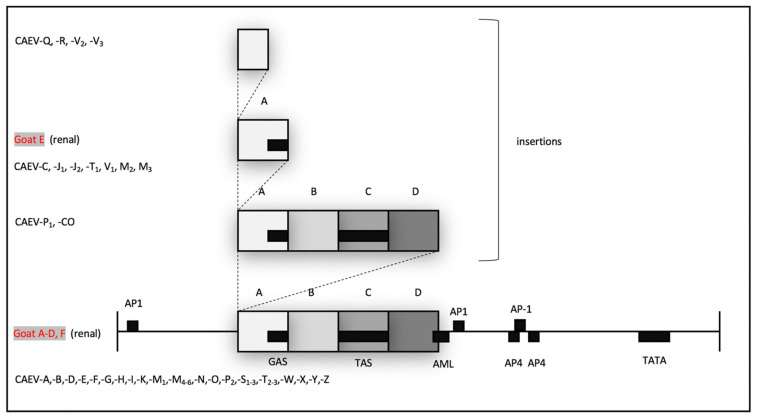
Schematic map of the CAEV U3 region (promoter). Promoter motifs are represented as black boxes and are named. The 70 base pair repeat (contiguous grey boxes) is broken into 4 subregions, A, B, C and D. Promoter isolates with sequence insertions are demonstrated schematically above the promoter map. CAEV sequences derived from renal isolates are in red (goats A–F); previously published sequences are in black (CAEV-A through CAEV-Z). Figure adapted with permission from BG Murphy, VM Elliott, N CVapniarsky, A. Oliver and J. Rowe (2010) [14].

Appropriate-sized PCR amplicons (CAEV U3 region, approximately 300 bp) were amplified using nested PCR from DNA samples isolated from the kidneys of all six goats and from the heart, lung and brain of goat D. An agarose gel electrophoresis image of the PCR amplicons derived from goat A is depicted in Figure 5. The complete CAEV U3 sequences determined from either direct sequencing of the PCR amplicons in both the 5′ and 3′ directions (goats A, D and E) or from cloned plasmids (goats B, C and F) are depicted in Figure 6 with the known transcription factor binding sites indicated by colored boxes. Goat B yielded a single CAEV cloned sequence, while goats C and F yielded three cloned sequences each from which a consensus sequence was determined. All of the CAEV U3 sequences have strong sequence preservation of the known transcription factor binding sites including the gamma-activated site (GAS), AML, AP-4 (2) and the TATA box. The TAS and AP-1 sites were less well conserved between the different amplicons. The U3 region isolated from goat E has an additional 17 nucleotide “A block” inserted as a tandem repeat in the proximal U3 region [14,17] containing a second 10 nucleotide GAS (Figure 6 and Figure 7). In the renal sequence derived from goat E, the two U3 A blocks are direct repeats and are separated by six nucleotides (CAGCAA).

**Figure 8:**
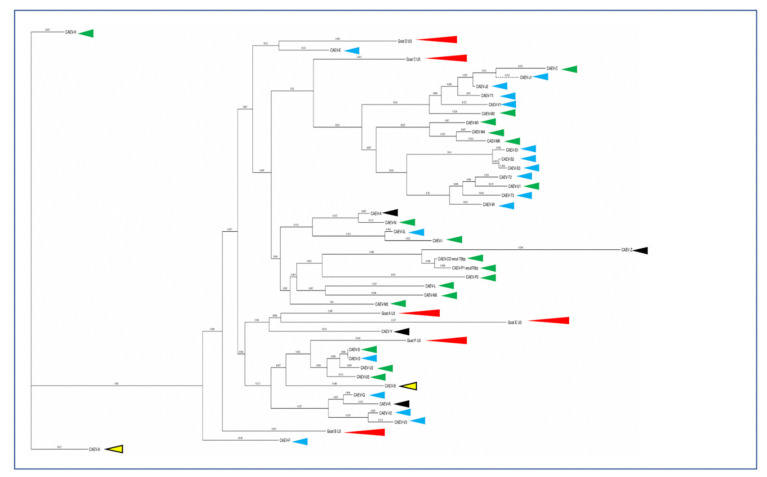
Phylogenetic dendrogram comparing the nucleotide sequences of CAEV U3 isolates from the renal tissue of goats A–E (red arrowheads) with previously published U3 sequences—tissues of the central nervous system (green arrowhead), joint synovium (black arrowhead), mammary gland (yellow arrowhead with black border) and lung (light blue arrowhead). CAEV A–Z sequences from [14]. Figure adapted with permission from BG Murphy, VM Elliott, N CVapniarsky, A. Oliver and J. Rowe (2010) [14].

A phylogenetic dendrogram is presented in Figure 8 depicting CAEV U3 sequences amplified primarily from Northern California goats since 2009. In this phylogenetic tree, the tissue of origin is encoded by red, light blue, dark blue, black or green arrowheads. The renal isolates from goats A and E cluster together while isolates from goats B, C, D and F do not.

## 4. Discussion

The clinical diagnosis of SRLV infections in small ruminants is typically based upon the identification of characteristic clinical signs of caprine arthritis, interstitial pneumonia, mastitis or disease of the central nervous system and then confirmed using serology. Renal and vascular diseases are not considered to be a typical clinical feature of CAEV infection, and renal lesions in goats infected with CAEV are rare. Here we describe renal interstitial nephritis lesions associated with amplifiable CAEV provirus in the kidneys of five dairy goats (Oberhasli, Nubian, Nigerian Dwarf and Alpine) and one non-dairy goat doe (Boer) with various multisystemic CAEV infections. Five of the six goats were serologically positive for CAEV antibodies; serum from goat F was not available for testing.

Although all of the animals had histological evidence of renal inflammation (interstitial nephritis), gross lesions in the kidneys were identified and reported in only three of the five goats. Gross renal lesions were identified as cortical infarcts (goat A) or multifocal pinpoint pale foci in either the cortex or medulla (goats B and D). Although the severity of the interstitial nephritis lesions varied markedly from animal to animal, all of the interstitial lesions featured a predominance of lymphocytes, with the lesions of some animals also demonstrating small numbers of plasma cells, neutrophils and/or the deposition of interstitial fibrous tissue. Lesion severity likely explains why the gross renal lesions were variably detected. One animal (goat B) had lymphoid follicle-like structures within the renal interstitium, a common histological feature in the lung and joint tissue of CAEV-infected animals. Inflammatory lesions in goat B were associated with tubular necrosis. In addition to the interstitial nephritis lesions identified in all of the goats, glomerular lesions were found in three animals (goats B, E and F). Mural arteritis lesions were identified in both the kidney and the myocardium of goat A associated with vascular thrombosis and tissue infarction. To our knowledge, this CAEV-associated lesion has not been reported previously in the peer-reviewed literature.

Although interstitial nephritis was identified in all of the goats, deposition of CAEV antigen was identified in only three animals by IHC (goats A, C and E). Viral antigen was also identified within the mural arteritis lesions of both the kidney and heart in goat A and within scattered medullary tubules (goats C and E) and endothelial cells (goat C). Whether the immunopositive material identified within the arteritis lesions represents smooth muscle cells or infiltrating macrophages was not determined. Although CAE viral RNA has been previously identified in the renal tubular epithelium [12], CAE viral antigen has not. The immunohistochemical detection of CAEV antigen remains a problematic aspect of CAEV diagnosis. Although the antibody utilized for this IHC assay is one of the antibodies used in the competitive ELISA serology test (VMRD), the sensitivity and specificity of the SRLV IHC assay have been questioned. Florid arthritis, mastitis and pneumonia lesions in CAEV-infected goats rarely demonstrate the presence of abundant CAEV antigen using IHC-based detection methods (authors’ personal experience). This may reflect limited viral antigen expression within the lesion or may be the result of assay insensitivity. The specificity of the IHC assay has also been questioned as pulmonary nematodes, intra-alveolar *Pneumocystis* organisms and caprine epithelial cells can stain as false positives with the CAEV IHC assay (authors’ personal experience). For these reasons, we confirmed the etiology using both serology and the tissue-specific presence of CAEV provirus using a nested PCR amplification strategy followed by sequencing.

Although CAEV is classically thought to be a monocyte/macrophage-tropic lentivirus, some studies have provided evidence that the viral host cell range may actually be broader. CAE viral transcripts have been identified in a wide variety of tissue types including the renal tubular epithelial cells, brain, spinal cord, lung, joint synovium, mammary gland, liver, spleen, lymph node, thyroid follicles and intestinal enterocytes [12]. Notably absent from this list of tissues are the smooth muscle cells of the arterial tunica media. It is important to acknowledge that this study does not allow the determination of whether or not these inflammatory lesions in the kidney and heart were actually caused by CAEV.

In these six CAEV U3 sequences, several of the transcription factor binding sites (GAS, AML, AP4 and TATA) are conserved, while other sites (AP1 and TAS) demonstrate some degree of sequence polymorphism. This pattern has been described previously for CAEV U3 sequences isolated from naturally infected goats [14]. The biological relevance of several of these sites has been previously explored [17,18,19]. The interferon gamma-mediated transcriptional activation of CAE viral transcription is mediated through the STAT1 pathway and requires the 10 nucleotide GAS within the CAE promoter [20]. The CAEV transcriptional response to IFN gamma has been shown to be proportional to the number of copies of GAS in the CAEV promoter [14]. The CAEV-Co isolate, first isolated and described in the 1970s and 1980s (Linda Cork and collaborators), has a conserved 70 base pair repeat (tandem direct repeat) containing the A block at the 5′ end. A naturally occurring CAE isolate encoding an identical 70 base pair repeat has only been described one other time, isolated from the central nervous system of a young Toggenburg goat from the University of Minnesota (CAEV-P1) [14]. However, 14 different CAE viral isolates encoding A block (or sub A block) repeats have been sequenced from the tissues of goats naturally infected with CAEV. Whether two copies of the GAS confer a selective advantage for viral replication (transcription) remains speculative. Although two of these renal isolates (A and E) form a phylogenetic cluster, the other renal isolates do not. As a result, there does not appear to be an association between the tissue of origin and the sequence of the CAEV U3 promoter. This study describes renal and arterial lesions in goats infected with CAEV, expanding the typical quartet of presentations of CAEV infection; however, the complete significance and viral causation of these lesions need further investigation.

## Figures and Tables

**Table 1:** Histological findings of goats A–E.

	Signalment	Serology	Kidney	Heart	Lung	Joints	Mammary	CNS
Goat A	6-year-old Oberhasli	+	PCR+, IN, TA, IHC+	MN, TA, IHC+, PCR−	NSF	CA IHC+	NE	E
Goat B	2-year-old Nubian mix	+	PCR+, IN, IHC−	MY	P	CA	LM	NSF
Goat C	1-year-old Alpine mix	+	PCR+, IN, IHC+	MY, VE IHC−	P	CA	LM	NSF
Goat D	6-year-old Boer mix	+	PCR+, IN IHC−	MN, PCR+	P, PCR+ IHC+	CA	LM	NSF, PCR+
Goat E	1.5-year-old Nigerian Dwarf	+	PCR+, IN IHC+	NE	P IHC+	NSF	NSF	NSF
Goat F	2-year-old Alpine mix	NE	PCR+, IN IHC−	NSF	P	NSF	NA	E,IHC+

IN = interstitial nephritis. LM = lymphocytic mastitis. TA = thrombotic arteritis. E = encephalitis. P = pneumonia. VE = valvular endocarditis. CA = caprine arthritis. IHC+/− = positive/negative for CAEV antigen by immunohistochemistry. MN = myocardial necrosis. NSF = no significant finding. MY = myocarditis. NA = not applicable. PCR+/− = pos/neg by PCR. NE = not examined. + = positive

## Data Availability

All of the study data is included in the manuscript and associated figures.
